# The influence of radiotherapy on ceruloplasmin and transferrin in whole blood of breast cancer patients

**DOI:** 10.1007/s00411-017-0708-3

**Published:** 2017-08-28

**Authors:** Ryszard Krzyminiewski, Bernadeta Dobosz, Tomasz Kubiak

**Affiliations:** Medical Physics Division, Faculty of Physics, Adam Mickiewicz University, Umultowska 85, 61-614 Poznań, Poland

**Keywords:** Electron paramagnetic resonance (EPR), Human blood, Radiotherapy, Breast cancer

## Abstract

Ceruloplasmin and transferrin are proteins which play a potential role in the process of breast cancer development. These molecules contain Cu^2+^ (ceruloplasmin) or Fe^3+^ ions (transferrin) and thus constitute paramagnetic centers, which can be studied using electron paramagnetic resonance method. The aim of the study was to determine how paramagnetic centers in whole blood of breast cancer patients change under the influence of radiation therapy. Samples of whole blood were taken from 17 women with breast cancer treated with radiotherapy. The measurements were carried out at 170 K using X-band electron paramagnetic resonance (EPR) spectrometer Bruker EMX-10. Two distinct EPR lines, derived from high-spin Fe^3+^ in transferrin and Cu^2+^ from ceruloplasmin, were revealed in all frozen samples. The amplitude and integrated intensity of the EPR signal from Cu^2+^ in ceruloplasmin significantly decreased in all patients after the delivery of the radiation fraction. When comparing the integral intensity of the signal from Fe^3+^ in transferrin, three different situations were identified which are patient specific: a significant increase, an insignificant change, or a significant decrease after the irradiation. A decreased level of Cu^2+^ from ceruloplasmin in patients after radiotherapy means a low level of ceruloplasmin in the plasma or an increased content of reduced Cu^+^ ions. Differences in the integrated intensity of the EPR signal from transferrin translate directly into the amount of bound iron. The observed changes could indicate how well the organism fights against cancer and how easily it adapts to the situation of biochemical stress.

## Introduction

Electron paramagnetic resonance spectroscopy is an ideal tool for the study of paramagnetic centers in human blood (Krzyminiewski et al. 2011; Kubiak et al. 2013). The centers include primarily the molecular complexes containing iron Fe^3+^ (transferrin, methemoglobin) or copper Cu^2+^ ions (ceruloplasmin). Changes in the paramagnetic centers can serve as a biomarker of breast cancer development because they reflect functional changes of ceruloplasmin and transferrin.

Human transferrin (Tf) is a protein that plays an important role because of its functions. The primary role of transferrin is to transport and deliver iron to the cells and it is also involved in the processes of cellular growth, proliferation, differentiation, and apoptosis. This protein constitutes a part of the defense system against excess free iron in the body. The presence of unbound iron leads, via the so-called Fenton reaction, to the formation of highly reactive hydroxyl radicals, which cause damage to lipids and the DNA (Liehr and Jones 2001). Transferrin delivers iron to actively dividing cells. It can play a role in the process of tumor growth, but this depends on the degree of cell differentiation (Gomme and McCann 2005).

Human Ceruloplasmin (hCP) is a glycoprotein present in the blood plasma. This glycoprotein has a molecular weight of 132 kDa (Bento et al. 2007; Farver et al. 1999; Zaitseva et al. 1996) and a carbohydrate content between 7 and 8% (Bento et al. 2007). In healthy adult people, its level is about 300 μg/ml (Healy and Tipton 2007). As a multifunctional enzyme it shows amino-oxidase, superoxide dismutase, and ferro-oxidase activity (Senra Varela et al. 1997). The oxidation of Fe^2+^ to Fe^3+^ by ceruloplasmin is necessary to load apotransferrin with iron. Human Ceruloplasmin (hCP) is an acute phase protein and antioxidant. It can also catalyze the reoxidation of Cu^+^ to Cu^2+^. Only Cu^2+^ ions can be studied by the method of electron paramagnetic resonance, because Cu^+^ ions are EPR silent (Hirota et al. 2000). The hCP protein contains six copper ions: three paramagnetic ions (Cu type I) in mononuclear sites and a trinuclear cluster with one paramagnetic (Cu type II) and two antiferromagnetically coupled ions (Cu type III) (Healy and Tipton 2007). Ceruloplasmin is synthesized in the liver, but it can be also produced by cancer cells. In the case of malignant tumors, the level of copper in the plasma (Zowczak et al. 2001) and the concentration of ceruloplasmin increase (Özyilkan et al. 1992; Senra Varela et al. 1997). In that situation, the rate of synthesis and secretion of this glycoprotein by the liver also increases. Tumor cells can capture a non-ceruloplasmin copper from plasma, so that they contain a relatively large amount of copper which has an effect on pathological angiogenesis (Zowczak et al. 2001).

The aim of present work was to identify paramagnetic centers in the whole blood of breast cancer patients and to determine how these centers change as a result of radiotherapy treatment.

## Materials and methods

The study material consisted of samples of whole blood which were collected from 17 women with breast cancer in the Greater Poland Cancer Center in Poznan. All patients suffered from carcinoma *ductale invasivum mammae*. The age of patients ranged from 30 to 68 years, with the average value of 55.5 years. Nine of them underwent breast-conserving surgery and eight-radical mastectomy. Besides histopathologically proven breast cancer, the anamnesis of the examined patients did not reveal any concomitant diseases that could affect the level of ceruloplasmin and transferrin in serum. Postoperative radiotherapeutic treatment was applied in all patients. Depending on the patient, the first irradiation (and first blood sampling) was performed between 15 and 197 days after the surgery. Generally, except for two cases (15 and 25 days), the first radiotherapy fraction was delivered more than a month after the operation. This relatively long period of time should reduce the possible influence of the surgery on paramagnetic centers present in blood plasma. The radiotherapy lasted for 21–30 days. External beam radiation therapy was used (photons with nominal energy of 6 MeV from a Varian Clinac linear accelerator). The dose of radiotherapy was individually chosen for each patient. They obtained from 17 to 25 dose fractions (usually 21 or 22) with 2.5 Gy per fraction. Radiotherapy was carried out every day for 5 days per week. 180 µl of peripheral blood was collected from each patient four times: directly before the start of the radiotherapy treatment, immediately after the first fraction (i.e., on the same day), directly prior to the last fraction and immediately after the last fraction (i.e., on the same day). Blood samples were transferred to EPR tubes (3 mm in diameter) and immediately frozen and stored in liquid nitrogen.

The EPR measurements were carried out using an X-band (9.4 GHz) Bruker EPR EMX-10 spectrometer equipped with the digital temperature control system ER 4131VT (temperature accuracy ±0.5 K). All spectra were recorded at 170 K temperature. The following settings were used: microwave power 20 mW, modulation frequency 100 kH, and modulation amplitude 1 mT. The EPR spectrum of each sample was registered in three magnetic field scan ranges: 650 mT (center 335 mT) for measurement of whole spectrum, 50 mT (center 335 mT) for measurement of Cu^2+^, and 50 mT (center 160 mT) for measurement of Fe^3+^. The standard weak pitch sample with concentration of free radicals equal to 2 × 10^13^ spins/cm of the sample tube was used to control the quality of the resonator. The relative concentration of paramagnetic centers in blood samples was evaluated as integrated intensity of the EPR spectrum. The integration was performed for appropriate EPR signals after a baseline correction.

## Results and discussion

**Fig. 1:**
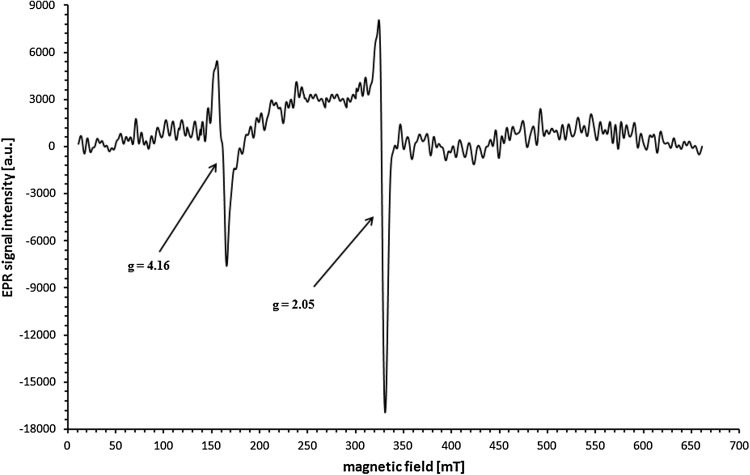
Typical EPR spectrum of whole blood from a breast cancer patient. The following *g* values are identified: *g* = 2.05 from Cu^2+^ ions in ceruloplasmin and *g* = 4.16 from Fe^3+^ ions in transferrin

An example of the EPR spectrum of whole blood of breast cancer patient is shown in Fig. 1. Two distinct EPR lines were detected in all frozen samples. Firstly, a line corresponding to *g* = 2.05 which corresponds to the literature value *g* = 2.049, which is assigned to Cu^2+^ ions in ceruloplasmin (Foster et al. 1973; Hirota et al. 2000; Pocklington and Foster 1977). The characteristic shape of the signal also confirms the origin of the line. Generally, it is the most intense component of the signal from Cu^2+^ in ceruloplasmin and probably comes from the Cu type II (Kouoh Elombo et al. 2000). Peak-to-Peak widths of the recorded signals of Cu^2+^ from ceruloplasmin are in the range of 5.3–7.5 mT.

**Fig. 2:**
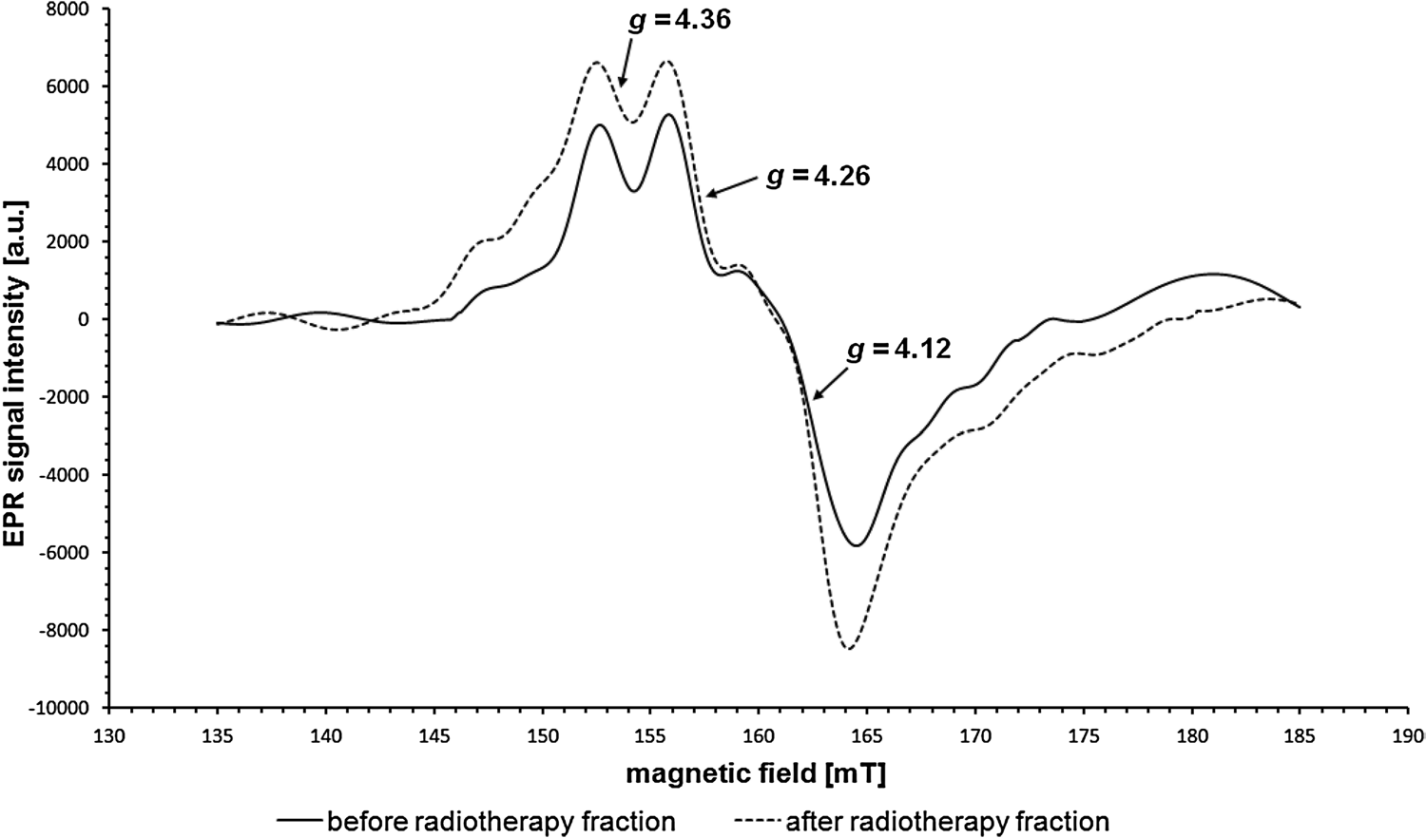
The increase in the integral intensity of the EPR signal from Fe^3+^ ions in transferrin after the delivery of the radiation dose. EPR signal in a patient before (*solid line*) and after (*dashed line*) radiation exposure

**Fig. 3:**
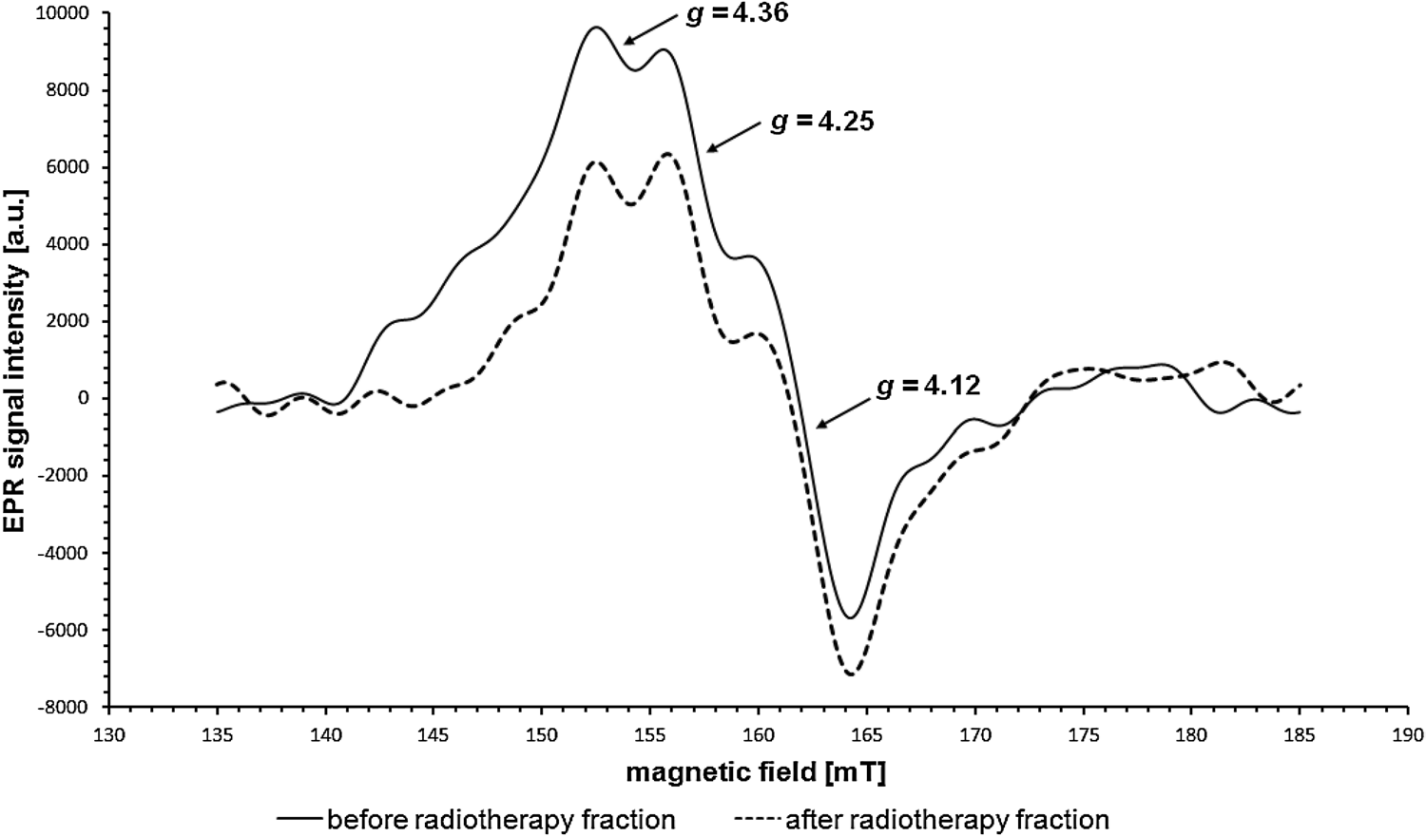
The decrease in the integral intensity of the EPR signal from Fe^3+^ ions in transferrin after the delivery of the radiation dose. EPR signal in a patient before (*solid line*) and after (*dashed line*) radiation exposure

The second of the recorded signals consists of three distinct components with different amplitudes. The *g* factor, assigned to the line formed by the merger of two components with highest amplitudes, is in the *g* range of 4.14–4.16. Comparison with the literature shows that this signal originates from Fe^3+^ in transferrin (*g* = 4.2) (Hirota et al. 2000; Kubiak et al. 2013; Pocklington and Foster 1977), which is also confirmed by the very characteristic shape. Two non-heme iron atoms are in the high-spin state *S* = 5/2 and their EPR spectrum is characteristic of the compounds in which iron is present in the rhombic symmetry system (Krzyminiewski et al. 2011; Pinkowitz and Aisen 1972) in an octahedral surrounding (Krzyminiewski et al. 2011). Because of the complexity of this signal, it has been recorded in the magnetic field range of 50 mT, which is shown in Figs. 2 and 3. The first component (counting in the direction of increasing values of magnetic field induction), for which *g* = 4.30–4.37, has the lowest amplitude and line width. The amplitude of the second component with *g* = 4.23–4.27 is greater than the amplitude of the first one. The third component, with *g* = 4.11–4.13, has the highest amplitude and greatest line width. The literature values of *g* factors for the three components are, respectively, *g* ≈4.39, *g* ≈4.19, and *g* ≈4.07 (Rottman et al. 1989).

**Fig. 4:**
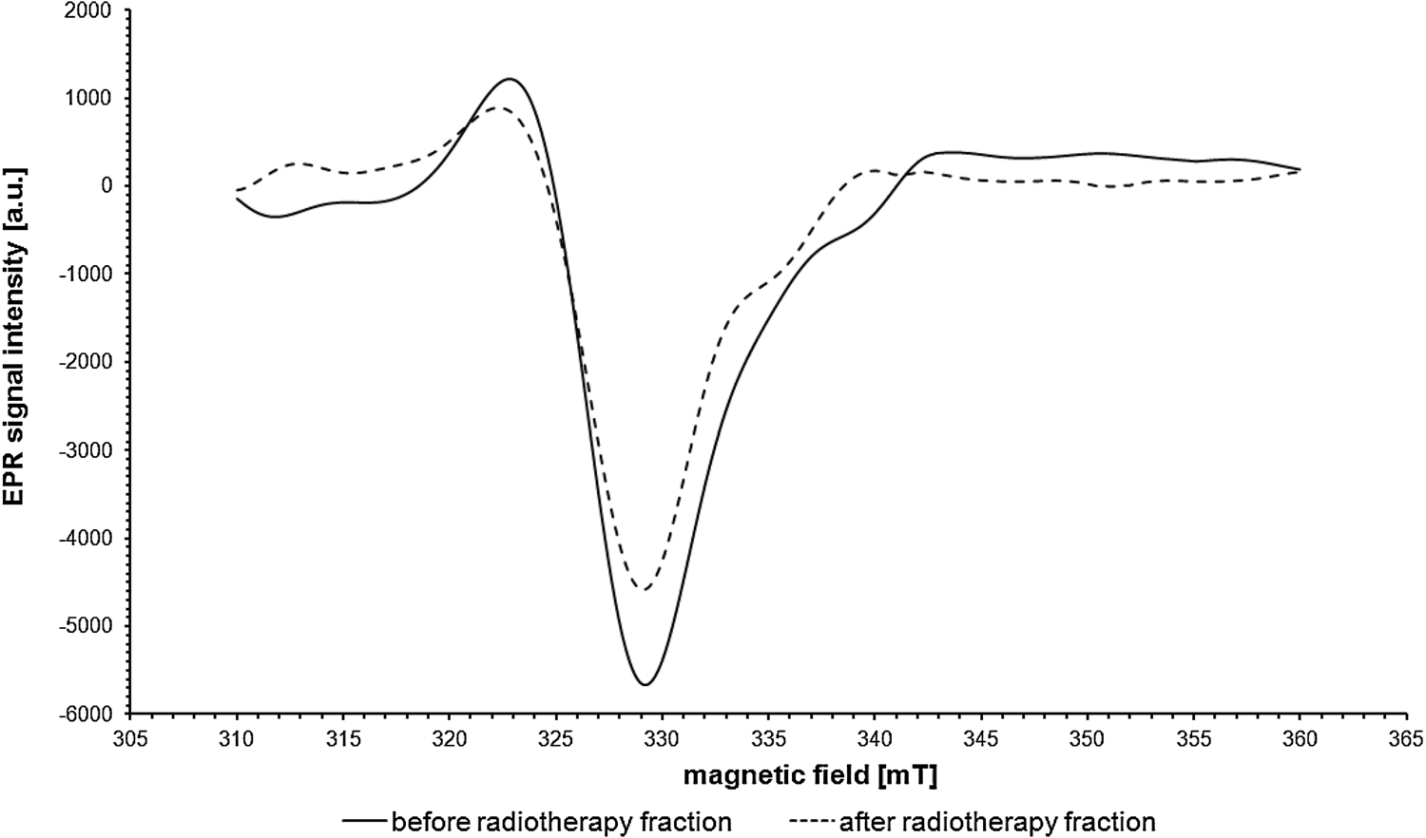
EPR signals from Cu^2+^ in ceruloplasmin in a patient before (*solid line*) and after (*dashed line*) radiation exposure

**Fig. 5:**
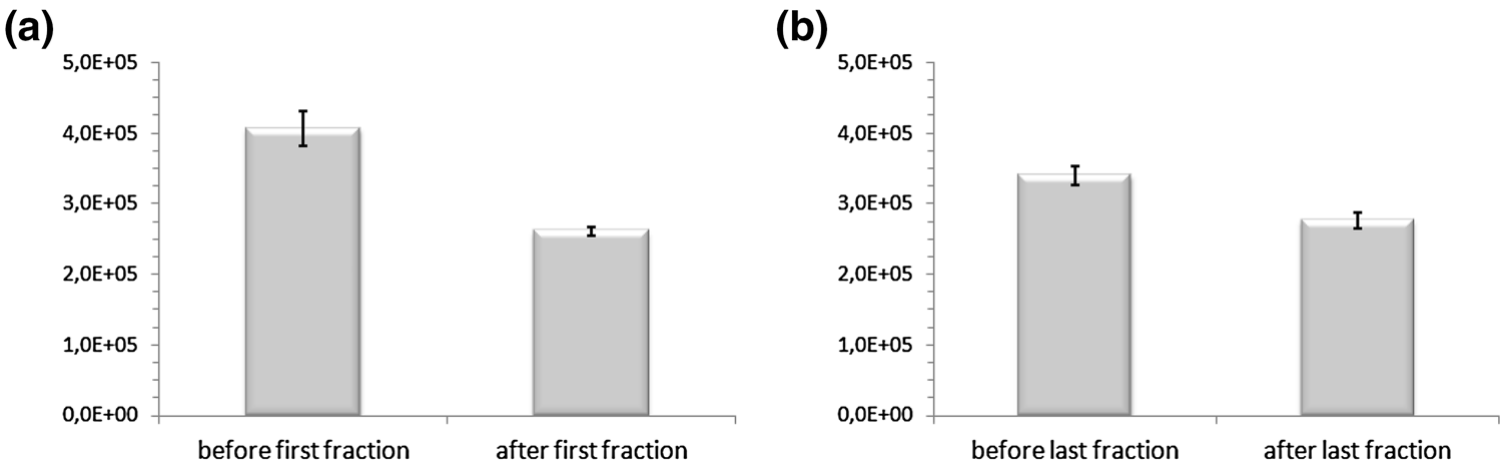
Mean values ± standard errors (SE) of the integral intensity of EPR signals from Cu^2+^ in ceruloplasmin: **a** before and after the delivery of the first fraction of radiation; **b** before and after the delivery of the last fraction of radiation

A comparison of EPR spectra from samples which were collected before and after the delivery of the single fraction of photon radiation was made for each patient. Figure 4 depicts the comparison of EPR signals from Cu^2+^ in ceruloplasmin in a patient before and after irradiation. There was a significant decrease in the amplitude and integral intensity of the signal from Cu^2+^ in ceruloplasmin in patients both after the delivery of the first and last fraction of radiation. After the first fraction, the decrease was seen in relation to the state before starting the treatment and after the last fraction it was seen in comparison with the situation prior to its delivery. It is worth noting that the decrease was stronger after the first fraction than after the last one. Table 1 shows that the mean values of the integral intensities of the EPR signals in patients before the delivery of particular fractions of radiation are higher than values after the delivery of these fractions (*p* < 0.05). These results with standard errors are graphically shown in Fig. 5. However, it should be noted that the mean integral intensity value of EPR signals from Cu^2+^ in ceruloplasmin measured before the delivery of the last fraction was 30% higher than the value determined after the first fraction. The last fraction resulted in a decrease of the mean integral intensity value by about 18% in relation to the state before this fraction. This is less than the 36% decline observed following the first fraction.

**Table 1:** Mean values, standard deviations (SD), and standard errors (SE) of the integral intensity values of EPR signals from Cu^2+^ in ceruloplasmin (17 patients)

Before first fraction			After first fraction			*p* value	Before last fraction			After last fraction			*p* value
Mean (a.u.)	SD (a.u.)	SE (a.u.)	Mean (a.u.)	SD (a.u.)	SE (a.u.)		Mean (a.u.)	SD (a.u.)	SE (a.u.)	Mean (a.u.)	SD (a.u.)	SE (a.u.)	
Cu^2+^ in ceruloplasmin													
4.1 × 10^5^	1.0 × 10^5^	0.24 × 10^5^	2.61 × 10^5^	0.25 × 10^5^	0.06 × 10^5^	<0.05	3.4 × 10^5^	0.5 × 10^5^	0.13 × 10^5^	2.8 × 10^5^	0.5 × 10^5^	0.11 × 10^5^	<0.05

Whatever the mechanisms, the declined Cu^2+^ signal response to radiation at the end of radiotherapy may reflect the progress and effectiveness of the treatment. A large signal decrease can be interpreted as an enhanced response of patient’s organism to the therapy. A reduced amount of Cu^2+^ does not necessarily mean less ceruloplasmin in the plasma, but only a high content of the reduced Cu^+^ form which is not detectable by EPR. A reduced level of ceruloplasmin or the presence of its inactive form is associated with a reduced enzymatic and antioxidant activity. Its reduced activity as a ferroxidase limits the iron binding by transferrin. Here, it is worth mentioning that transferrin has only the ability to bind Fe^3+^ ions.

Some studies provide examples of the impact of radiation on the ceruloplasmin in human plasma. In the Chernobyl disaster liquidators, the content of inactive ceruloplasmin in blood significantly increased after 2 weeks of exposure to ionizing radiation (Pulatova et al. 2009). Studies published by Swartz and Wiesner (1972) showed increased EPR signals from ceruloplasmin in cancer patients and decrease of this signal in response to radiation therapy. Tomas et al. (1972) described a decrease in serum level of Cu after exposure to ionizing radiation. Patients with head and neck cancer had significantly elevated serum ceruloplasmin level as compared to controls and this increase was directly proportional to the stage of cancer (Sachdeva et al. 1993). After radiotherapy, a significant decrease in ceruloplasmin level was observed, but the value was still higher than in the control group.

If the increase of ceruloplasmin concentration is caused by the response of the organism to oxidative stress and reflects the proper functioning of natural adaptive mechanisms, then high doses of ionizing radiation, which intensify this stress through lipid peroxidation, should result in a further increase of this protein in plasma. However, in the case of radiotherapy-treated breast cancer patients, there was a decrease in the amount of Cu^2+^ ions in ceruloplasmin after the delivery of radiation and this translates into a reduction of protein function. This effect may indicate an excessive strain of the body and the gradual depletion of its defense capabilities. On the other hand, it may also reflect the body’s response to therapy, which aims to eliminate the tumor.

**Fig. 6:**
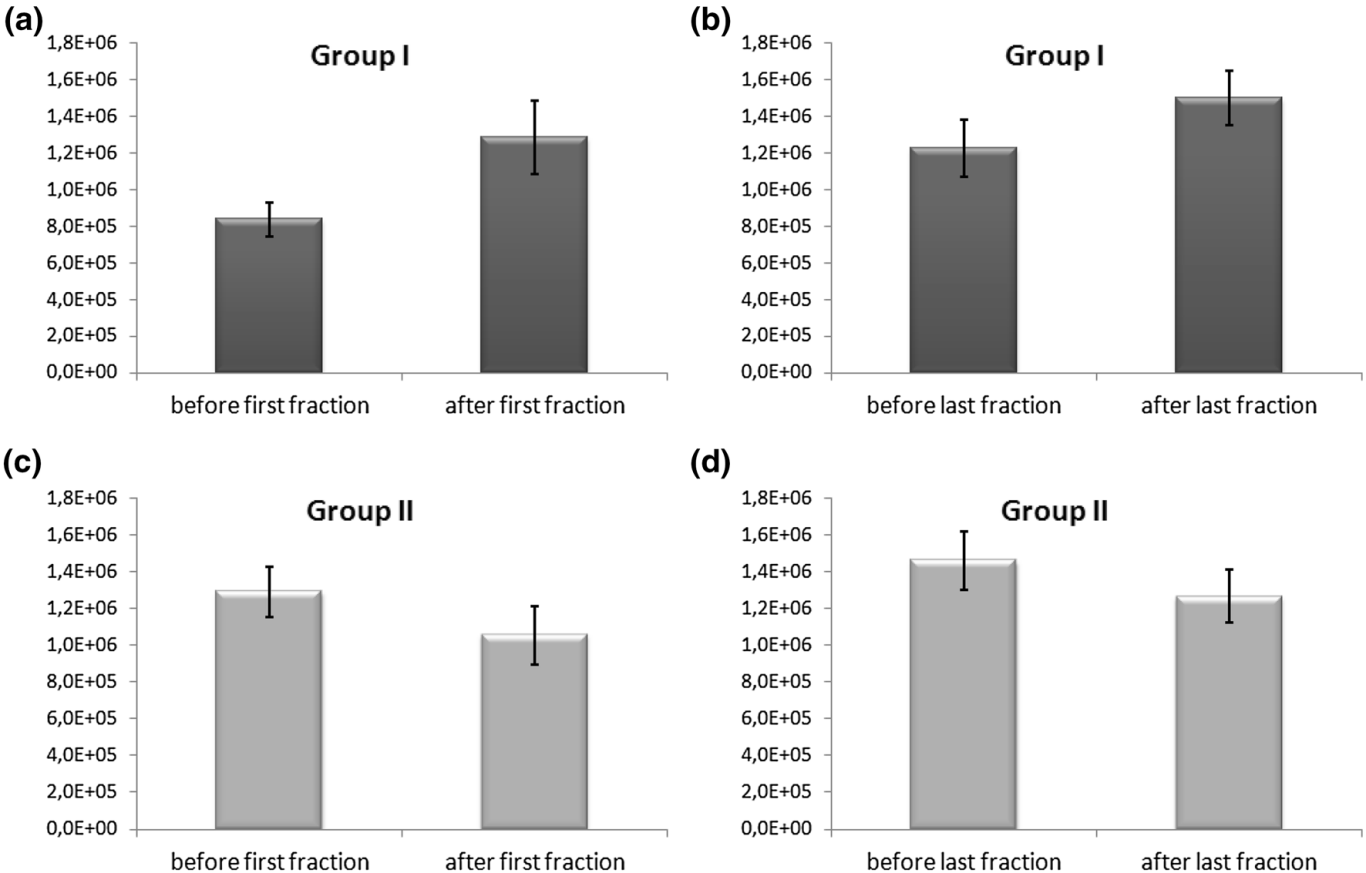
Mean values ± standard errors (SE) of the integral intensity of EPR signals from Fe^3+^ in transferrin in patients from group I (**a**, **b**) and group II (**c**, **d**)

Changes in integral intensities before and after radiation exposure are also visible in case of EPR lines from Fe^3+^ in transferrin. Three patient-specific scenarios can be identified: (1) a significant increase in the integral intensity of the signal after radiation exposure; (2) insignificant changes; and (3) a significant decrease in the integral intensity. Examples are shown in Figs. 2 and 3. Also, a comparison was made of the mean values of integral intensities of EPR signals in patients with an increased signal after the delivery of radiation (group I—5 people) and in patients with a decreased signal (group II—8 people). The results are shown in Table 2 and, with marked standard errors, in Fig. 6. Insignificant changes were observed in case of 4 individuals.

**Table 2:** Mean values, standard deviations (SD), and standard errors (SE) of the integral intensity values of EPR signals from Fe^3+^ in transferrin in patients from group I (5 patients) and group II (8 patients)

Before first fraction			After first fraction			*p* value	Before last fraction			After last fraction			*p* value
Mean (a.u.)	SD (a.u.)	SE (a.u)	Mean (a.u.)	SD (a.u.)	SE (a.u)		Mean (a.u.)	SD (a.u.)	SE (a.u)	Mean (a.u.)	SD (a.u.)	SE (a.u.)	
Group I: increase in Fe^3+^ from transferrin (5 patients)													
0.84 × 10^6^	0.21 × 10^6^	0.09 × 10^6^	1.3 × 10^6^	0.5 × 10^6^	0.2 × 10^6^	<0.05	1.22 × 10^6^	0.35 × 10^6^	0.16 × 10^6^	1.5 × 10^6^	0.33 × 10^6^	0.15 × 10^6^	<0.05
Group II: decrease in Fe^3+^ from transferrin (8 patients)													
1.29 × 10^6^	0.39 × 10^6^	0.14 × 10^6^	1.1 × 10^6^	0.5 × 10^6^	0.16 × 10^6^	<0.05	1.5 × 10^6^	0.5 × 10^6^	0.16 × 10^6^	1.3 × 10^6^	0.4 × 10^6^	0.14 × 10^6^	<0.05

A statistical analysis of the data from patients from group II revealed a significant decrease (*p* < 0.05) of the mean integral intensity value of EPR signals from Fe^3+^ in transferrin both after the delivery of the first fraction of radiation (18% decline vs before the exposure) and after the delivery of the last fraction (13% decline vs before the exposure). However, it should be noted that the mean value measured before the delivery of the last radiation fraction was 38% higher than the value determined after the first fraction. A decreased integral intensity of a signal from Fe^3+^ in transferrin means less amount of iron bound to this protein. The direct reason for this situation is probably a decreased availability of Fe^3+^ due to a reduced ceruloplasmin function as ferroxidase. Carrying out the oxidation of Fe^2+^ to Fe^3+^ is indeed necessary to load apotransferrin with iron. It is worth mentioning that the integral intensity of the EPR signal from Cu^2+^ in the ceruloplasmin decreased after the irradiation, suggesting a decrease in the level of this protein in whole blood or converting it into an inactive form. A declining level of saturated transferrin during radiotherapy treatment may result in increased oxidative stress due to a high level of free iron in the bloodstream. This situation is detrimental to the organism because the presence of protein-unbound iron can lead, through the so-called Fenton reaction, to the formation of reactive oxygen species (ROS) such as hydroxyl radicals. Some authors have noted a significant decrease in the level of transferrin in cancer patients treated by radiotherapy (Gomme and McCann 2005; Koc et al. 2003). A reduction in the amount of transferrin-bound iron was also observed in the Chernobyl disaster liquidators after 2 weeks of exposure to ionizing radiation (Pulatova et al. 2009).

Group I includes those patients who had a significant increase of the integral transferrin signal intensity after radiotherapy. This situation applies to five women. Here, the increase after the delivery of the first fraction (53% vs before the exposure) was greater than the increase observed after delivery of the last fraction (23% vs before the exposure). The rise in the amount of transferrin-bound iron may be associated with a reduced transfer of this metal to cells, which would result in an increased level of transferrin compared with apotransferrin. Under normal conditions, only about 30% of transferrin binding sites are occupied by iron ions (Sanna et al. 2009), thus additional uptake of Fe^3+^ ions could occur as an effect of the body’s response to oxidative stress caused by radiation exposure. In the literature one can find results of studies carried out using EPR, which show that the serum level of Fe^3+^ bound to transferrin increases linearly with the received radiation dose (Pulatova et al. 2009). Also, elevated levels of iron in transferrin were observed in blood samples of children living in areas of high radioactive contamination with ^137^Cs (Pulatova et al. 2009). Thus, the increase of the transferrin pool may be regarded as a biochemical marker of adaptation of the organism and show how well the organism fights against cancer and how easily it adapts to the situation of the biochemical stress.

At the end it is worth recalling once more that the EPR signal from Fe^3+^ ions is related to the saturated form of transferrin (holoprotein). Apotransferrin is EPR silent (Dunne et al. 2006), therefore the EPR measurements do not reflect the total transferrin content in plasma. Similarly, EPR is able to show the amount of Cu^2+^ ions in ceruloplasmin, not the whole concentration of this protein in plasma.

## Conclusion

Studies have shown changes in the paramagnetic centers in whole blood of patients with breast cancer under the influence of radiotherapy treatment. Firstly, there is a significant decrease in the amplitude and integrated intensity of the signal from Cu^2+^ in ceruloplasmin after the delivery of the fraction of radiation, which translates into less ceruloplasmin in the plasma or higher content of reduced Cu^+^ ions. Secondly, in the case of the signal from the Fe^3+^ from transferrin, depending on the patient, a significant increase in the integrated intensity of the signal after delivery of the radiation dose (higher amount of iron bound to the proteins), insignificant changes, or a significant decrease in the integrated intensity (less amount of bound iron) after delivery of the fraction takes place.
